# Transient Psychotic Relapse Following COVID-19 in a Stable Schizophrenia Patient on Paliperidone Palmitate: A Case Report

**DOI:** 10.7759/cureus.74661

**Published:** 2024-11-28

**Authors:** Syed Ali Bokhari, Mariam J Karaman, Madhusudan Deepak Thalitaya

**Affiliations:** 1 Psychiatry, Al Amal Psychiatric Hospital, Emirates Health Services, Dubai, ARE; 2 Psychiatry, Maudsley Health, Al Amal Psychiatric Hospital, Dubai, ARE; 3 Psychiatry, Mohammed Bin Rashid University of Medicine and Health Sciences, Dubai, ARE

**Keywords:** antipsychotics, coronavirus infectious disease, covid-19, psychiatry, sars-cov-2 (severe acute respiratory syndrome coronavirus-2), schizophrenia

## Abstract

Schizophrenia is a chronic psychiatric disorder marked by severe disturbances in thought, perception, and behavior. Long-acting injectable (LAI) antipsychotics, such as paliperidone, are widely used to promote sustained remission and ensure medication adherence, especially in patients prone to relapse. However, the COVID-19 pandemic has introduced unique challenges, with studies indicating that infections like COVID-19 may exacerbate psychiatric symptoms through neuroinflammatory pathways. This report presents the case of Ms. X, a 48-year-old Emirati female patient with chronic schizophrenia, who achieved long-term stability on paliperidone LAI at 150 mg monthly. For almost two years, she remained stable with notable improvements in delusional and paranoid symptoms, supported by structured therapeutic care, supportive psychotherapy, occupational therapy, and family involvement. However, in August 2024, Ms. X contracted COVID-19, which triggered a sudden relapse marked by acute persecutory delusions, heightened suspicion, and avoidance behavior, despite ongoing adherence to her LAI regimen. Following her recovery from COVID-19, Ms. X’s psychotic symptoms remitted without changes to her antipsychotic treatment, and she returned to her baseline functioning. This case highlights the sensitivity of chronic schizophrenia to physiological stressors such as viral infections, underscoring the importance of vigilant monitoring and comprehensive care during periods of illness. It also contributes to the growing body of evidence suggesting that infections can induce transient yet significant psychiatric relapses in otherwise stable schizophrenia patients, emphasizing the intricate interplay between physical and mental health. This case underscores the complex interplay between physical and mental health in schizophrenia and highlights the need for further research into infection-induced psychiatric exacerbations.

## Introduction

COVID-19, first reported to the World Health Organization (WHO) in December 2019, rapidly escalated into a global pandemic by March 2020. Its rapid spread led to unprecedented socio-economic and psychological impacts worldwide, as individuals and communities faced lockdowns, remote working conditions, and restricted movement. This new reality, combined with a pervasive fear of infection, precipitated widespread mental health challenges across all demographics. Meta-analyses revealed elevated levels of anxiety, depression, post-traumatic stress, and insomnia among both the general public and COVID-19 patients, with neuropsychiatric manifestations, including new-onset psychosis, observed in some cases [1,2].

Schizophrenia is a chronic and often debilitating mental illness characterized by disturbances in thought, perception, and behavior, significantly impacting patients’ lives. Among available therapies, long-acting injectable (LAI) antipsychotics, such as paliperidone, have shown considerable efficacy in promoting remission and stability in schizophrenia patients. However, the COVID-19 pandemic has posed unique challenges for patients with chronic schizophrenia, particularly in maintaining mental health stability [1,3].

Globally, the pandemic’s effect on mental health systems has been varied, with notable increases in hospitalizations for psychotic episodes in several countries [4]. In contrast, regions such as the UK and Italy observed reductions in psychiatric admissions during initial lockdowns, possibly due to fears of infection in healthcare settings [5,6]. The interplay between viral infections, immune responses, and schizophrenia symptomatology has garnered growing interest, as COVID-19 infection appears to exacerbate psychiatric symptoms in some patients, leading to psychotic relapses even in those previously stable on antipsychotic regimens [2].

In the United Arab Emirates, COVID-19’s onset resulted in an increase in psychiatric admissions, as a single tertiary psychiatric facility absorbed all psychiatric admissions in Dubai and the Northern Emirates, with other facilities temporarily closed. This period also saw a higher prevalence of new psychiatric cases without previous histories [2].

Amid these trends, individuals with chronic mental illnesses, such as schizophrenia, were disproportionately impacted [7,8]. Schizophrenia patients face unique risks from viral infections due to immune dysregulation, cognitive deficits affecting preventive behavior adherence, and higher comorbidities like diabetes, which may complicate COVID-19 outcomes [9]. Reports indicate that stress, fear, and social isolation related to the pandemic often exacerbate schizophrenia symptoms and elevate relapse rates, even in patients stable on antipsychotic regimens [10].

This report presents the case of a chronic schizophrenia patient who experienced a transient psychotic relapse during a COVID-19 infection, despite stable treatment with long-acting paliperidone. The patient returned to remission once the infection resolved, highlighting the need for focused strategies to support this vulnerable population during infectious outbreaks.

## Case presentation

Ms. X, a 48-year-old Emirati female patient with a well-documented history of schizophrenia, was initially diagnosed in 2017 at the psychiatry department of a general medical facility. Her presenting symptoms included persecutory delusions, auditory hallucinations described as derogatory voices criticizing her actions, and disorganized thinking characterized by incoherent speech and difficulty maintaining logical connections in her thoughts. Over the years, her illness trajectory involved five psychiatric admissions due to medication non-compliance and functional decline.

In 2018, Ms. X was transferred to our hospital, a tertiary psychiatric facility, where she underwent a comprehensive reassessment. This included psychological testing to observe her behavior and cognition during her ward stay, social evaluations to assess family dynamics, and a medical workup, including complete blood count, liver function tests, C-reactive protein, thyroid function tests, and electrolyte levels. On presentation to our facility, she was on oral aripiprazole 5 mg once daily, which offered only partial symptom relief. She was experiencing persistent paranoia, impaired self-care, and episodic aggression. Following this, her treatment was optimized. Thereafter, she was admitted.

At the time of her initial admission, Ms. X was prescribed oral risperidone, starting with 2 mg nightly, which was eventually titrated to 6 mg nightly. Oral risperidone was later transitioned to paliperidone palmitate LAI 150 mg every four weeks. This choice was based on prior non-adherence to oral medications and paliperidone’s efficacy in managing psychotic symptoms in patients with schizophrenia who exhibit poor insight.

Between 2019 and 2022, Ms. X’s trajectory involved periods of symptomatic stability interspersed with treatment adjustments due to persistent challenges. During this time, she experienced recurring difficulties with treatment adherence, leading to episodic functional decline and behavioral disturbances. In 2022, following a period of symptomatic instability, she was reinitiated on paliperidone LAI 150 mg monthly at the same dose. The consistent therapeutic levels provided by the LAI allowed her to achieve prolonged stability, maintaining remission for nearly two years without any signs of psychotic relapse. During this period, from September 2022 onward, she showed significant progress: her paranoia and delusional thinking completely abated, she consistently engaged in self-care routines, and she actively participated in occupational therapy sessions.

Her continued admission as a long-stay inpatient was influenced by social factors, including unresolved divorce proceedings, financial instability, and a lack of adequate family support and housing. However, she developed a reliable support network with her family, particularly through regular visits from her brother and daughter, which reinforced her treatment adherence and provided additional emotional support.

Her stable period continued uninterrupted until August 2024, when Ms. X contracted COVID-19. The diagnosis was confirmed through reverse transcription polymerase chain reaction (RT-PCR), with a positive test result for SARS-CoV-2. Clinical symptoms included an initial fever of 38.6°C with nasal congestion but no respiratory distress or other significant symptoms. Initial laboratory findings included a white blood cell count of 7.12 x 10³/mcL, platelet count of 268 x 10³/mcL, hemoglobin level of 12.8 g/dL, and elevated C-reactive protein of 29.7 mg/L.

On the third day of her infection, Ms. X began exhibiting a sudden relapse of her psychotic symptoms. Despite remaining on her usual paliperidone LAI 150 mg monthly regimen, she developed acute persecutory delusions, characterized by intense fears of external threats and heightened suspiciousness. She expressed beliefs that her healthcare providers were conspiring against her and attempting to harm her through her medications. She also articulated fears that the virus was intentionally implanted in her to monitor her thoughts and actions. This period was marked by withdrawal from staff and family, refusal to eat or take medications due to fears of poisoning, and hypervigilance.

The treating team did not add additional oral medications during this critical period as her symptoms, while acute, did not necessitate further pharmacological intervention. There were no contraindications, and nursing care adhered strictly to infection control protocols. Symptomatic treatment for COVID-19 included paracetamol for fever, with no drug-drug interactions identified that could explain the psychotic symptoms. Delirium was ruled out based on the absence of fluctuating consciousness, disorientation, or other hallmark features of delirium. Furthermore, Ms. X was able to recall her experiences during the episode, further supporting that her symptoms were psychotic in nature rather than delirium.

Recovery from COVID-19 was determined based on the resolution of symptoms, including the absence of fever and flu-like symptoms, such as nasal congestion, sore throat, or cough, by the fifth day of isolation. Vital signs returned to normal, with a consistent temperature of 36.7°C, respiratory rate of 18 breaths per minute, oxygen saturation at 99% on room air, and blood pressure of 132/74 mmHg. A chest X-ray conducted on the fifth day revealed clear lung fields, no pleural abnormalities, and an unremarkable mediastinal and thoracic structure. Follow-up diagnostic testing confirmed a negative RT-PCR result, indicating the resolution of the active SARS-CoV-2 infection.

Following her recovery, Ms. X’s psychotic symptoms progressively began to remit without any adjustments to her treatment plan, as her persecutory delusions diminished, and her baseline cooperative and stable behavior reemerged. She resumed her engagement in structured therapeutic activities, reestablished her self-care routines, and reconnected with her family, who continued to visit regularly.

Ms. X remains admitted to the hospital as a long-stay inpatient and continues on her paliperidone LAI regimen of 150 mg monthly. Her ongoing treatment plan emphasizes stability maintenance, with regular psychiatric evaluations conducted within the inpatient setting to monitor for any signs of potential relapse. She has resumed her full participation in therapeutic programs and occupational activities.

## Discussion

The COVID-19 pandemic has led to an increased interest in understanding the association between viral infections and psychiatric exacerbations in schizophrenia patients. Various studies have highlighted that individuals with schizophrenia are at higher risk for severe COVID-19 outcomes due to both physiological and behavioral factors. For instance, schizophrenia patients often have impaired immune responses and cognitive deficits that may affect their adherence to preventive health measures, contributing to a higher risk of infection [8,11]. Moreover, emerging research has linked viral infections, including COVID-19, with neuroinflammatory responses that could trigger or exacerbate psychotic symptoms [1,12].

The pathophysiology underlying COVID-19-induced psychiatric exacerbations is thought to involve multiple mechanisms, including neuroinflammation, immune dysregulation, and potential interactions with psychotropic medications. SARS-CoV-2, the virus responsible for COVID-19, has neuroinvasive capabilities, leading to an inflammatory response within the central nervous system (CNS). This inflammatory cascade may result in heightened levels of cytokines, which are known to affect neurotransmitter systems, potentially triggering psychotic symptoms or reducing the efficacy of antipsychotic medications [3,11]. This hypothesis is supported by studies indicating increased relapse rates in patients on stable antipsychotic regimens who develop COVID-19, possibly due to altered drug metabolism or immune modulation effects induced by the virus [7,10].

The stress associated with the pandemic, including social isolation and fear of infection, has also been identified as a significant factor contributing to psychotic relapses among schizophrenia patients. Studies have shown that elevated anxiety and fear levels associated with COVID-19 correlate with increased symptom severity in schizophrenia, with spirituality acting as a potential moderating factor to mitigate these effects [1]. This emphasizes the importance of psychosocial support during the pandemic, especially for schizophrenia patients, to help manage anxiety and prevent relapse.

Regarding treatment, LAI antipsychotics, such as paliperidone, have been recommended to maintain stability in schizophrenia patients during the pandemic due to their efficacy and reduced need for frequent healthcare visits. Despite their benefits, reports indicate that a significant number of patients have still experienced relapses during the pandemic, with studies documenting increased relapse rates due to both physiological and psychological impacts of COVID-19 [13]. Additionally, adherence to LAIs was challenged during the pandemic, as disruptions in healthcare services limited access to regular appointments, contributing to lapses in treatment and subsequent relapses [14,15].

Another hypothesis proposes that COVID-19 infection may interfere with the pharmacodynamics or pharmacokinetics of antipsychotic medications. For example, studies have documented cases where patients on clozapine experienced a significant psychotic episode during COVID-19 infection, despite continued adherence to treatment, suggesting that the virus may alter drug response [3]. COVID-19-induced neuroinflammation may impact the blood-brain barrier or alter drug metabolism pathways, possibly diminishing the effectiveness of antipsychotic drugs. This could explain why some patients, including the patient in this case, may relapse during an active COVID-19 infection yet return to remission as soon as the infection resolves, indicating that remission may be achievable as the neuroinflammatory effects of the virus subside [11].

## Conclusions

This case highlights the susceptibility of chronic schizophrenia patients, even those in long-term remission on long-acting antipsychotic therapy, to relapse during COVID-19 infection. Ms. X’s abrupt psychotic relapse, characterized by persecutory delusions during her COVID-19 infection, underscores the potential for physiological stressors, such as viral illness, to disrupt mental health stability in schizophrenia. Her return to baseline mental state post-infection reinforces the need for vigilant monitoring and supportive care during such stress periods.
